# The effectiveness of intervention based on the transactional model on improving coping efforts and stress moderators in hemodialysis patients in Tehran: a randomized controlled trial

**DOI:** 10.1186/s12882-021-02592-8

**Published:** 2021-11-11

**Authors:** Mohtasham Ghaffari, Mohammad Ali Morowatisharifabad, Mohammad Saeed Jadgal, Yadollah Mehrabi, Somayeh Alizadeh

**Affiliations:** 1 Environmental and Occupational Hazards Control Research Center, School of Public Health and Safety, Shahid Beheshti University of Medical Sciences, Tehran, Iran; 2 Health Department, School of Public Health, Yazd Shahid Sadoughi University of Medical Sciences, Yazd, Iran; 3 Tropical and communicable diseases research center, Iranshahr University of Medical Sciences and health services, Iranshahr, Iran; 4 Department of Public Health, School of Nursing and Midwifery, Iranshahr University of Medical Sciences and Health Services, Iranshahr, Iran; 5 School of public health and safety, Shahid Beheshti University of Medical Sciences, Tehran, Iran; 6 Health Department, School of Public Health, Kerman University of Medical Sciences, Kerman, Iran

**Keywords:** Coping efforts, Moderators, Education, Transactional model, Hemodialysis

## Abstract

**Background:**

Present study was conducted to determine the effect of training on coping efforts and stress moderators, based on transactional model of Lazarus and Folkman, in hemodialysis patients.

**Methods:**

This is a randomized controlled clinical trial on 116 hemodialysis patients referred to dialysis centers in Tehran from May to August 2018. The patients were assigned to two experimental and control groups using a simple randomization method. The intervention included 6 training sessions in the form of coping efforts and moderators of transactional model. Data were collected before and 3 months after the intervention. Data were analyzed using SPSS 16.

**Results:**

After 3 months training intervention, there was significant increase in the intervention group in the mean scores of coping efforts (*P* < 0.001), moderators and subscales of emotional regulation from 51.18 ± 20.42 to 64.87 ± 13.18 (P < 0.001), dispositional coping style from 45.56 ± 19.45 to 55.84 ± 18.03 and social support from 49.61 ± 20.14 to 55.55 ± 17.35 (*P* < 0.005).

**Conclusion:**

The training based on transactional model was successful in the increase of social support, dispositional coping style and emotional regulation in hemodialysis patients. Therefore, Nurses and healthcare providers can use this program to help hemodialysis patients to increase their adaptation to the illness and reduce stress.

**Trial registration:**

IRCT registration number: IRCT20180524039814N1; Registration date: 13-08-2018; Registration timing: retrospectively registered: Last update: 13-08-2018.

## Introduction

Chronic renal failure is a global public health problem which has raised many concerns worldwide [1]. Patients with end-stage renal disease (ESRD) experience a lot of changes in their lives. These changes are due to their dependence on hemodialysis apparatus, which is associated with many difficulties such as physiological and psychological-social challenges [2]. According to previous research, this disease has doubled in 1990–2010. It was also classified among 18 causes of death worldwide in 2010 [3]. Dialysis is the common treatment for chronic renal failure, and this disease is unique in terms of treatment since the only way to postpone the death is tracking of this debilitating disease [4]. The effects of chronic renal failure, lack of treatment, and the effect of the treatment on lifestyle and welfare are considered as sustainable stress sources in the lives of these people [5, 6]. Thus, hemodialysis can be associated with many stressors.

Lazarus and Folkman believe that stress is a relative concept of a complex and dynamic interaction between an individual and the environment. The ways or strategies that a person uses in dealing with stressful situations play an essential role in their physical and mental health, and the individual’s vulnerability is associated with understanding stress and its sources [7, 8]. The transactional model of Lazarus and Folkman is one of the methods organizing the ways which people adapt to chronic illness [9, 10].

According to the transactional model of stress when a person is exposed to a stressor, the first stage is the primary appraisal. In this stage, the person internally determines the severity of the stressor. If at this stage the stressors are perceived threatening, a secondary appraisal is performed, in which the person evaluates own resources to deal with stress [11]. Performing appraisals by individuals influences the coping strategies chosen by them [1]. The main premise of the transactional model is that primary appraisal, secondary appraisal, and coping strategies mediate between stressors and the consequences of stress in individuals so that individuals choose and apply the appropriate coping style based on interaction with others and their living environment [11].

**Fig. 1:**
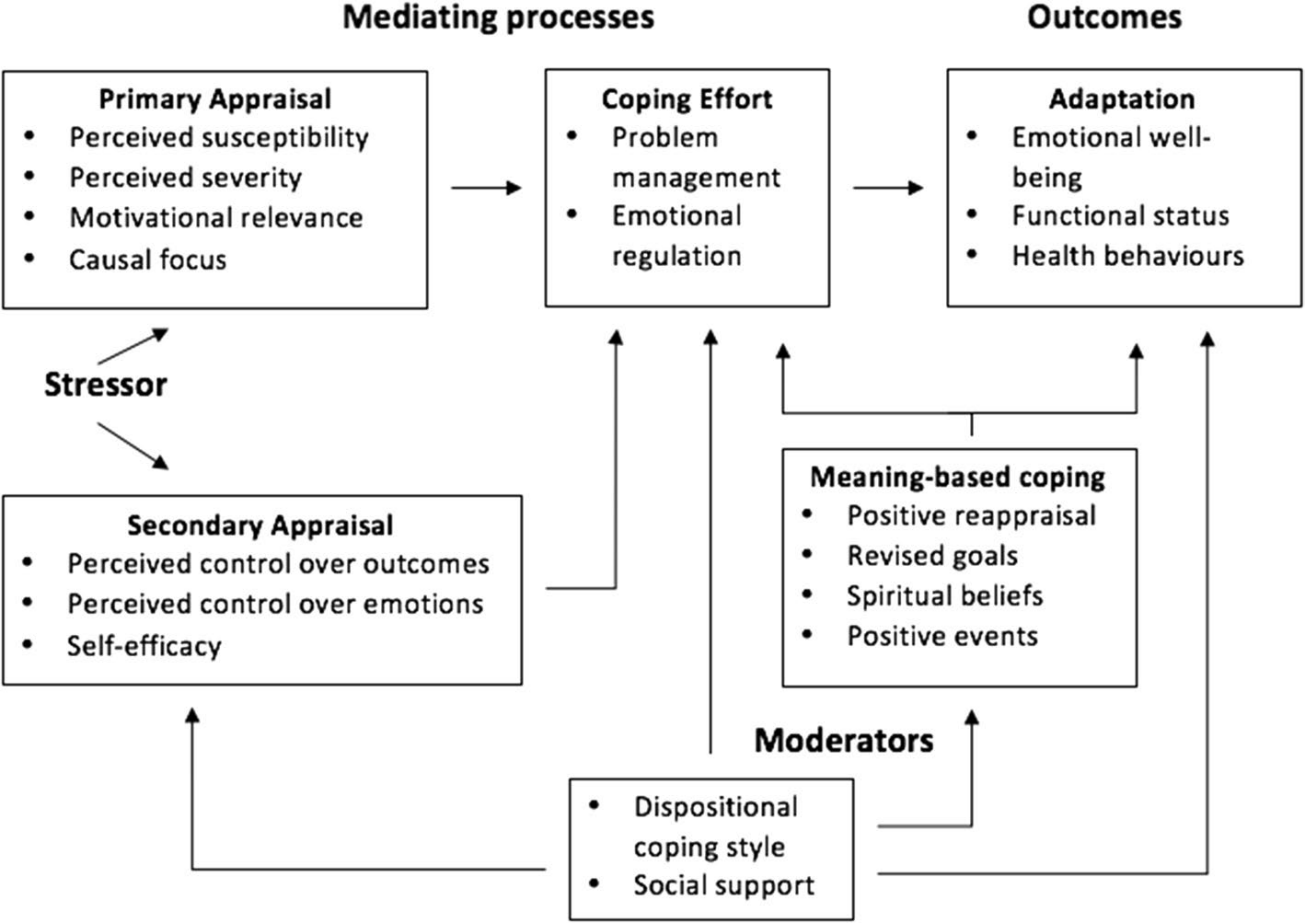
Transactional model of stress and coping

According to Lazarus and Folkman, there are two types of coping strategies. The problem- focused coping strategy tries to eliminate or alter the source of stress, while emotional regulation coping strategy is a way to deal with a stressor focusing on altering the way one thinks or feels about a situation or something [12]. The structures and substructures of the transactional model are shown in Fig. 1.

During stressful situations, individuals may mostly use one of these two types of coping styles or even use both strategies at the same time. Note that there is no good or bad coping strategy, and people will use each of the coping methods according to their situation. In coping efforts, there may also be a constructive or non-constructive mode [13]. Therefore, the use of one style does not necessarily mean that one must use a constructive strategy when faced with stressors in his/her life. In the transactional model, the moderators signify the ability to search for information as well as build and maintain relationships with others, which involves information search and social support [14]. The transactional model of Lazarus and Folkman is well-known as one of the most effective patterns in stress management [11]. So far, there have been only a few studies examining the effectiveness of interventions based on the Lazarus and Folkman’s model. The results of a study showed that both patterns reduced stress among teachers, where the transactional model of stress and adaptation was more effective compared to the usual education program [15]. Another study indicated that multifaceted treatment of Lazarus had a significant effect on the general health. Also, ANCOVA test showed that multifaceted treatment significantly reduced depression, anxiety, and social function disorder [16]. Another researcher reported that the educational program based on the transactional model reduces the level of stress and encourages the use of healthy coping styles in Multiple Sclerosis (MS) patients [10]. Further, the role of multiple stress management interventions, based on the transactional model, in improving adaptation responses and stress score among students was confirmed in one the study [17].

Dialysis patients require special attention in terms of coping efforts, social support, and dispositional coping styles.

In a study conducted by the authors of this article, the results showed that.

In a study conducted by the authors of this article, the results showed that after intervention base on transactional model mean score of primary appraisal and substructures (perceived susceptibility, motivational relevance, casual focus) and secondary appraisal and substructure of self-efficacy had improve. So transactional model of stress could improve the correct appraisal of hemodialysis patients for stressful situations [9]. In previous study, only how to appraise stress in hemodialysis patients has been studied, while paying attention to coping styles and moderators in hemodialysis patients is also very important to reduce stress in hemodialysis patients. On the other hand the review of literature also revealed no studies assessing the effect of coping efforts and stress moderators training in hemodialysis patients. It seems that further studies including educational programs using appropriate models are necessary to help patients in terms of mental relaxation and rational behavior when faced with stressors resulting from the disease in order to promote the health of hemodialysis patients. Accordingly, the present study was conducted to determine the effect of training on coping efforts and stress moderators, based on the transactional model of Lazarus and Folkman, in hemodialysis patients in Tehran.

## Materials and methods

### Study design and setting

This is a single-blinded, randomized controlled trial conducted from May to August of 2018 in Resalat Dialysis Center and Partian Dialysis Center in Tehran, Iran.

### Participants

The study population included all patients undergoing hemodialysis in Tehran. The simple randomization method was used to select two dialysis centers from Dialysis Centers of East of Tehran which were closely related in terms of social, cultural, and economic characteristics. The city of Tehran, as the capital of Iran, has the largest population, so there are cultural, economic and social differences in the city. But usually people living in an area in terms of income level, access to municipal services, level of social participation, religious affairs, social class, food habits, type of religion (although the predominant religion in Iran is Shiite, but in the study area some people they had Armenian and Zoroastrian religions) on the same level. Therefore, when two dialysis centers are selected from the same area, people are very similar in terms of these characteristics.

Then, one of the dialysis centers was randomly assigned to the intervention group and the other to the control group. The centers were separated from each other such that the intervention group could not provide information to the control group after which the sample was randomly selected from among them.

### Sample size

In order to determine the sample size, considering the error of the first type 5%, the test power was 90% with 95% confidence with respect to the mean and standard deviation values of stress score as 36.2 ± 9.47 and 64.12 ± 17.64 according to previous studies [18], the sample size was *n* = 52 in each group. Finally, with a 10% drop in the study, the sample size was set as *n* = 116 where each of the intervention and control groups had *n* = 58.

$$\boldsymbol{n}=\frac{{\left({\boldsymbol{Z}}_{\mathbf{1}-\frac{\boldsymbol{\alpha}}{\mathbf{2}}}+{\boldsymbol{Z}}_{\mathbf{1}-\boldsymbol{\beta}}\right)}^{\mathbf{2}}\left({\boldsymbol{S}}_{\mathbf{1}}^{\mathbf{2}}+{\boldsymbol{S}}_{\mathbf{2}}^{\mathbf{2}}\right)}{{\left(\boldsymbol{\Delta }\right)}^{\mathbf{2}}}$$

$${Z}_{1-\frac{\alpha }{2}}=1.96\ {\left({Z}_{1-\frac{\alpha }{2}}+{Z}_{1-\beta}\right)}^2=10.5$$

$${Z}_{1-\beta }=1.28\ \left(\mu 2-\mu 1\ \right)={\left(\Delta \right)}^2$$

Mean in the first community (*μ*1) = 36.2.

Mean in the second community (*μ*2) = 64.12.

Standard deviation in the first community $\left(\ {S}_1^2\right)$ = 9.47.

Standard deviation in the second community $\Big({S}_2^2$) = 17.64.

**Fig. 2:**
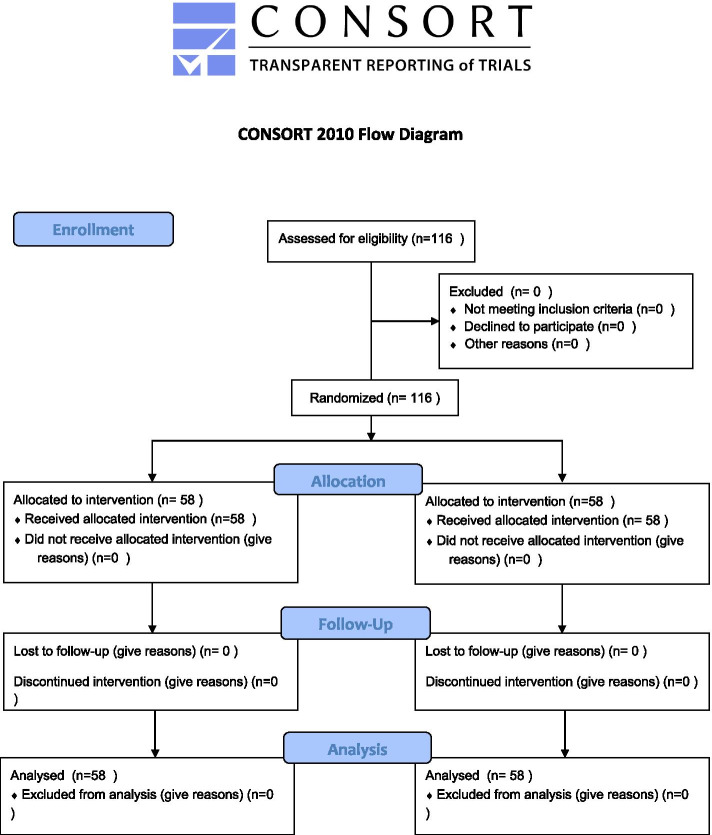
CONSORT 2010 Flow Diagram

The study inclusion criteria were: chronic kidney disease, Tehran residence, hemodialysis, having a file at the dialysis center, having at least reading and writing skills, ability to attend training sessions, and having no history of chronic psychological disease. On the other hand, the study exclusion criteria included psychiatric treatment, reluctance to participate in the study, and having a history of relevant training. Flow diagram of entering subjects in the study groups has shown in Fig. 2.

### Data collection

A researcher-made questionnaire was used to collect data prepared based on the transactional model of Lazarus and Folkman. The questionnaire consisted of demographic questions and 33 questions for measuring the coping efforts and moderators of the transactional model. The number of questions in each subscale included the following cases: 21 questions that measure coping efforts and include 8 questions in problem management (I study relevant journals to overcome the stress resulting from disease) and 13 questions in the emotional regulation (as I’m not able to do anything, I calm down myself with sedative drugs) and 12 questions that measure moderators and include 6 questions in the social support (I talk to my doctor about the stresses of the disease and want him/her to help me), and 6 questions in the dispositional coping style (I try to respond to the stress in the best way). The questions 1–21 were scored according to 3-point Likert scale as (1) agree, (2) no idea (3) disagree. On the other hand, questions 22–33 were scored based on 4-point Likert scale as (1) never (2) rarely (3) sometimes, (4) always. The scores ranged from zero to 100. The scoring method was as follows: higher score in the problem management and the emotion-focused coping indicated weaker problem management and emotional regulation, respectively. Similarly, higher scores in the social support and the dispositional coping style indicated less social support and no change in the dispositional coping style under different conditions.

### Content validity

Content Validity is a method of assessing the validity of a questionnaire. Content validity answers questions such as: Does the tool item measure what it should? Does the designed tool include all the main aspects of the concept of measurement? Validity of qualitative content, such as grammar and use of appropriate words was consulted with the panel of experts (including 8 health education and promotion specialists, 2 psychologists and 2 kidney specialists). After collecting expert evaluations, the necessary changes in the tools was made in consultation with the members of the research team.

Based on the number of experts who evaluated the questions, the minimum acceptable Content Validity Ratio (CVR) value is determined based on the Laosche table. Questions that the amount of CVR calculated for them is less than the desired amount according to the number of evaluators should be excluded from the questionnaire. Given the number of evaluators in our study was 12, the Laosche number is 0.56. Questions whose CVR value was less than 0.56 were excluded from the test and thus scored 96 items higher than Laosche’s number (0.56). The value of Content Validity Index (CVI), 0/85, indicated acceptable instrumental validity.

### Reliability

There are several methods for measuring the internal consistency of the questionnaire, one of the most common of which is the Cronbach’s alpha coefficient, which is based on the mean covariance (or correlation) of the questions in a questionnaire. When there are several subscales in the questionnaire, alpha is calculated separately for the subscales. The reliability of the tool was measured using Cronbach’s alpha method, which was as follows: problem management (0.75), emotional regulation (0.77), social support (0.86), and dispositional coping style (0.71).

In the next step, construct validity was performed using exploratory factor analysis. The statistical population of the study at this stage included hemodialysis patients.

The sample size suggested for factor analysis is 100 poor, 200 fair, 300 good, 500 very good and 1000 excellent [19]. Also in confirmatory factor analysis and structural model, the minimum sample size is determined based on hidden variables that 20 samples are required for each hidden factor or variable (in this study the hidden variables are the dimensions of the model) and in general at least 200 samples are recommended [20]. Therefore 340 hemodialysis patients were selected by an available sampling method.

In research based on exploratory factor analysis, determining the sample size and the existence of appropriate correlations between variables are of particular importance. Kaiser-Meyer-Olkin (KMO) in factor analysis allows researchers to make sure that the sample size is adequate. And Bartlett test is used to test homogeneity of variances before performing factor analysis.

The KMO test was used for the adequacy of the sample size and the Bartlett Test of Sphericity was used to evaluate the suitability of the collected data for factor analysis. Based on the obtained results, KMO (0.87) and the amount of chi-square calculated for Bartlett sphericity test 15,552.1 were obtained which are statistically significant (*P* < 0.001). Based on these results, the data are suitable for factor analysis Table 1.

**Table 1:** KMO and Bartlett’s test of Sphericity

KMO	Bartlett’s test of Sphericity		
Sample adequacy	x^2^	df	Sig
0.87	15552.1	1890	P <0.001

In order to confirm the structure obtained from exploratory factor analysis, confirmatory factor analysis was performed. As shown in Table 2, the Chi-square index with its degree of freedom was less than 5. Also, GFI, NFI and CFI gain indices of more than 0.9 and RMSEA less than 0.08 were obtained, which confirmed the fitness of the model.

**Table 2:** fit index of transactional model

χ2/df	RMSEA	GFI	RMR	NFI	CFI
2.35	0.049	0.95	0.092	0.91	0.92

It should be noted that the researcher-made questionnaire included all the structures and substructures of the transactional model with 96 questions, which due to the extensiveness of the model, some of the results have been published in another article [9]. Therefore, in this article, a part of the questionnaire and its results are given.

The measurement tool was administered to both the intervention and control groups before and 3 months after the intervention through which the data were collected.

### Intervention

After obtaining approval from the Ethics Committee and Vice-Chancellor for Research of Yazd University of Medical science, the researcher referred to the dialysis centers and obtained permission from their officials. The researcher then gave clear explanations of the study methodology and objective for the participants and answered their concerns and questions then informed consent was obtained from them.

At the intervention center, as the patients were convenient to be trained in the center of dialysis, all of them were trained in the center, but the questionnaire was only available to the participants in the study. The intervention group was divided into several 5 or 6-individual groups who received 6 sessions of training as lecture, group discussion, and question and answer. Each group received one training session per week. The training sessions were conducted by the corresponding author of the article, a psychologist and a religious cleric and the average duration of each session was about 2 h.

The first session included: inauguration, expression of goals, and discussion topics, general matters about stress, and stress and illnesses. The second session covered relaxation exercises, exercises to relieve stress, stretching, contraction and expansion of muscles, exercise of self-relieving, deep breathing, progressive relaxation training, and training mental imagery exercise, where the patients were asked to do these exercises each night for 15–20 min before going to the bed. Also, 15 min of each session was devoted to the relaxation exercises in the subsequent sessions. The patients also received educational pamphlets with the trained subjects. The third session involved definition of coping, types of coping, definition of problem management, training problem management, training the use of problem solving to deal with stressful situations (acceptance of situation, precise definition of problem, prioritizing problems, precipitation of thoughts, decision-making, implementing and evaluating the results). The fourth session included the definition of emotional regulation, training skills to control emotions, the correct way to express emotional reactions, training strategies for increasing body potential to deal with stress, and training emotional regulation methods. The fifth session worked on explaining the dispositional coping style and how it changed, definition of interpersonal relationships, importance of interpersonal relationships, definition of social support, types of social support, asking for help from others, and coping with loneliness. Also, patients’ families received educational pamphlets during this meeting which included introducing the most common stressors in dialysis patients and how to deal with mental and psychological problems in these patients to enhance the social support. Finally, the sixth session covered reviewing the presented topics, practicing the discussed topics, employing training sessions for adaptive coping, reviewing negative thoughts and how to challenge them, as well as reviewing and explaining individual patient achievements. After completing the training sessions, a booklet containing all the materials presented during the sessions was given to the patients.

### Data analysis

Data were analyzed using SPSS 16 software. Kolmogorov-Smirnov test and graphical methods were used to check the normality of data distributions. Paired t-test was utilized to compare the scores obtained before and 3 months after the intervention. Also, independent samples t-test was used to compare the scores between the two groups 3 months after the intervention. Comparison of the scores obtained 3 months after the intervention between the two groups, while adjusting them for baseline values was performed by analysis of covariance (ANCOVA). Significance level for all statistical tests was set at *P* < 0.05.

### Ethical considerations

Before the intervention, the objectives of the course were explained to the participants, and informed consent was obtained from them. Ethical code: R.SSU.SPH.REC.1397.012.

Also study was registered at clinical trials registration center with the code of IRCT ID: IRCT20180524039814N1

## Results

The mean age of participants in the study was 52.81 years old with standard deviation of 7.71. The minimum age was 22 years and the maximum age was 70 years old. Other demographic information of the participants is presented in Table 3. The intervention and control groups were similar in terms of demographic variables at the beginning of the study and did not have any significant difference (Table 3).

**Table 3:** Comparison of participants’ demographic variables in the intervention and control groups

Variable	group	intervention group		control group		ϰ^2∗^	***P***-value
		Frequency	percentage	Frequency	percentage		
gender	male female	29	50	31	53.4	0.7	**0.42**
		29	50	27	46.6		
Marital status	Married Single Widowed	45	82.8	44	75.9	1.31	**0.51**
		5	8.6	5	8.6		
		5	8.6	9	15.5		
Education	Elementary intermediate High school Higher education	12	20.7	13	22.4	0.32	**0.95**
		17	29.3	15	25.9		
		16	27.6	18	31		
		13	22.4	12	20.7		
Employment status	Employed housewife Retired Self-employed	11	19	13	22.4	7.01	**0.22**
		19	32.8	19	32.8		
		18	31	14	24.1		
		10	17.2	12	20.7		
History of the disease in the family	yes no	10	17.2	7	12.1	0.008	**0.78**
		48	82.8	51	87.9		

Comparing the mean scores of coping efforts and its subscales before and three months after the intervention between two groups, the mean scores of coping efforts and emotional regulation between two groups, indicated a significant increase in the intervention group (*P* < 0.001). According to the results of covariance analysis, in which the difference between groups was adjusted for the baseline value of each score, a significant improvement was observed in the coping efforts and emotional regulation scores 3 months after in the intervention group compared to the controls. The score of emotional regulation before the intervention in the intervention group was 51 ± 20, which reached 64 ± 13 3 months after the intervention. Also, the total score of coping efforts before the intervention in the intervention group was 52 ± 19, which increased to 60 ± 13 after the educational intervention. While in the control group, no statistically significant difference was observed before and after the educational intervention (Table 4).

**Table 4:** Comparison of coping effort scores between intervention and control groups before and three months after intervention

Variable	group	before intervention	three months after intervention	^*^P-value
		Mean ± SD	Mean ± SD	
Emotional regulation	intervention	20.42 ± 51.18	13.18 ± 64.87	<0.001
	control	17.56 ± 54.84	16.12 ± 55.06	0.43
	P^**^	0.3	0.001	
	P^***^		<0.001	
Problem management	intervention	20.44 ± 54.41	17.66 ± 56.88	0.22
	control	18.74 ± 55.71	18.78 ± 55.06	0.15
	P^**^	0.72	0.59	
	P^***^		0.19	
Coping effort	intervention	19.73 ± 52.8	13.76 ± 60.87	0.001
	control	17.19 ± 55.28	16.30 ± 55.33	0.92
	P^**^	0.9	0.04	
	P^***^		<0.001	

P^*^: paired t-test, P^**^: t-test, P^***^: ANCOVA

Regarding the score of moderators and its subscales, significant changes were seen in the intervention group after 3 months. Specifically, the scores of the moderators and subscales of social support and dispositional coping style showed a significant increase 3 months after the intervention. The score of social support before the intervention in the intervention group was 49 ± 20, which reached 55 ± 17 3 months after the intervention (*P* < 0.005). Dispositional coping style score also increased significantly in the intervention group from 45 ± 19 to 55 ± 18 (*P* < 0.001). Also, the total score of the moderators before the intervention in the intervention group was 49 ± 14, which after the educational intervention reached 55 ± 14 (P < 0.001). On the other hand, while the average scores of the moderators and its subscales had a relative improvement in the control group, these changes were not statistically significant (Table 5).

**Table 5:** Comparison of moderators’ scores between intervention and control groups before and three months after intervention

Variable	group	before intervention	three months after intervention	^*^P-value
		Mean ± SD	Mean ± SD	
Social support	intervention	20.14 ± 49.61	17.35 ± 55.55	0.005
	control	21.84 ± 48.08	18.74 ± 48.56	0.7
	P^**^	0.78	0.03	
	P^***^		0.006	
Dispositional coping style	intervention	19.45 ± 45.56	18.03 ± 55.84	<0.001
	control	17.80 ± 48.85	15.75 ± 47.6	0.37
	P^**^	0.93	0.01	
	P^***^		<0.001	
moderators	intervention	14.40 ± 49.09	14.40 ± 55.69	<0.001
	control	17 ± 48.46	13.92 ± 48.08	0.73
	P^**^	0.83	0.004	
	P^***^		<0.001	

P^*^: paired t-test, P^**^: t-test, P^***^: ANCOVA

## Discussion

The present study is one of the few studies in which an educational program based on coping efforts and moderators of transactional model was used to enhance compatibility and reduce stress in hemodialysis patients.

In this study, there was a significant increase in emotional regulation in the experimental group after the educational intervention, indicating that the patients were able to use more healthy emotional regulation strategies after the intervention, and the use of unfavorable coping strategies had diminished in these individuals. Undesirable coping strategies can disrupt the overall performance of the patients with chronic hemodialysis. Taheri et al. found that the hemodialysis patients mostly use emotional regulation strategies to cope with the challenges [21]. In agreement with the present study, the results of another study showed that the mean scores of emotional regulation in the experimental group were altered, indicating the effectiveness of the intervention [13]. Another researcher also noted the effect of educational intervention on the reduction of emotional regulation score, which was carried out on women with back pain [22]. In another study it was observed that the hemodialysis patients mostly use emotional regulation strategies to cope with the stress [23]. Many studies suggest the effectiveness of educational intervention in improving the use of coping styles in different groups and diseases, which are in line with the present study [24–27]. Psychological intervention based on emotional regulation made patients successful in managing stress. It seems that teaching techniques based on emotional regulation by encouraging people to practice frequently and focus on the body and mind can relieve stress patients from mental occupation. During the interventions, patients learned to deal with negative emotions and thoughts, to experience mental events positively, and to have proper control over the stresses caused by their illness.

In the present study, the mean problem management score had a nominal increase and decrease in the intervention and control groups, respectively. However, these changes were not statistically significant.

Other research results showed that the patients with chronic renal disease tended to less use direct coping styles compared with their healthy counterparts and were more likely to use avoidance and relieving coping styles in dealing with stress in their lives; thus, they tend to use more emotional regulation [28]. A study on MS patients showed that the problem management is not related to mental health in patients with multiple sclerosis [29]. Probably, the patients with MS disease are similar to those undergoing hemodialysis due to the burden of disease, challenge or impotence, occupational problems, and costs of treatment. Thus, it may be concluded that the patients with chronic diseases as well as prolonged and severe treatment courses consider their condition as an uncontrollable problem, where consequently the problem management loses its effectiveness. As a result, the inadequacy of the present research in improving the problem management may originate from the same reasons. As Lazarus and Folkman stated in their transactional model, individuals who experience uncertainty and evaluate conditions uncontrollable, use emotional regulation strategies [30]. It should be reminded that there is no superiority between the emotion-focused or the problem-focused coping styles to be used, but employing a problem management is combined with more self-control and more self-efficacy [31].

The coping efforts in the experimental group improved in comparison with the control group after the intervention, indicating the effectiveness of educational intervention in improving the use of coping efforts in the hemodialysis patients.

In the present study, the moderators included subscales of the dispositional coping style and social support.

The dispositional coping style is defined as general ways of behavior which can affect emotional response or individual performance to a stressor. In a research entitled “relationship between the positional and dispositional coping styles”, defined these two styles as the following: the positional coping style is a stable strategy that one usually uses in all stressful events, while the positional coping style is a special coping strategy used by the person in a special stressful condition [32]. The mean score of the dispositional coping style increased significantly in the test group after the intervention indicating that the patients mostly used various coping styles adapted to specific stressful situations. A recent study suggested that personality had the greatest variance with positional and dispositional coping, but their relationship was different, where cognitive assessments gave increased credibility to the positional coping, outside of the compatibility aspects [33]. Another study stated that the dispositional coping styles in childhood may be influenced by the first childhood experience. Also, regression analysis revealed that an emotion-focused coping style can be predicted by negligence and emotional abuse during childhood [34]. In another study the results indicated that project managers use coping strategies and active planning in stressful situations, and a high level of organizational performance is related to greater use of planning coping strategies [35]. In a study, the researchers found that people with alcohol abuse, according to the dispositional coping style, use avoidance coping styles compared to healthy coping styles. Further, in terms of gender differences in the dispositional coping style, women used coping styles more to have a positive interpretation of stressful situations, while men tended to be more religious [36]. It seems that one of the reasons that patients improved their dispositional coping style is that we tried to teach them to apply a specific coping strategy to a particular stressful situation and the correct methods of information seeking during the training sessions. Information seeking is acquiring information and knowledge in order to appraisal the risk and correctly identify the stressor. The unknowns can make the coping process difficult. Because when a person does not have enough information about the stressor, he cannot have a proper appraisal of the stressful situation and the amount of health threat [11]. Since ambiguity is always existing in healthcare settings, information seeking is one of the coping responses that is frequently used in healthcare settings.

The social support addresses individual efforts for obtaining information, tangible and emotional support, such as sharing your emotions and problems about a stressful event with others, receiving help from expert and prominent individuals, asking for a respected person’s opinion and advice, accepting sympathy and others’ confidence to solve the problem, and effective coping with the stressful situation [17].

The comparison of the average social support score in the test and control group before and after the intervention indicated that this score increased significantly in the test group, suggesting that social support improved in the patients after the intervention. Participation of the families of patients and medical staff to enhance social support in the patients was one of the positive aspects of this study. Some studies are also consistent with the present study [13, 22]. A previous study implied that social support plays a major role in chronic diseases, and the individuals with more social support have higher flexibility for chronic pain [37]. The role of peer support groups on self-transcendence in patients undergoing hemodialysis was emphasized in past study [38]. Another study also reported that increased social support leads to enhanced psychological dimension of life quality in hemodialysis patients [39]. Findings of another study pointed to a significant relationship between all areas of social support and death anxiety in hemodialysis patients [40].

In another study, Sadoughi et al. revealed a significant relationship between perceived social support and quality of life in patients on hemodialysis. They found that there is an inverse relationship between quality of life and anxiety as well as depression in dialysis patients [41]. A study demonstrated the direct relationship between social support and quality of life as well as the survival rate of hemodialysis patients [42]. In the present study, the situation that group training provided to express the excitement and problems and concerns caused by the disease among similar people, was very effective in receiving social support from their peers. On the other hand, in the present study, we tried to provide interventions to increase social support from the family for patients because receiving emotional support from the family has a significant effect on the disease process. The support that the patient receives from family, friends and important people in his life can help the person to cope and better adapt to the complications of the disease.

Despite its robustness by including adequate sample size and random sampling, the present study had a few limitations, including the statistical population which merely involved patients on hemodialysis. This certainly limits the generalizability of findings and interpretations of the cognitive causes in other diseases which should be considered. Also, since many dialysis patients are undergoing peritoneal dialysis, it is suggested that studies on stressors and adaptation methods in patients undergoing peritoneal dialysis should also be conducted in future. Finally, the stressor factors and adaptive methods among the two groups of patients undergoing the hemodialysis and the peritoneal dialysis should also be compared.

## Conclusion

This study suggested that the educational intervention based on the transactional model is helpful in improving coping efforts and moderators in hemodialysis patients. Training using appropriate educational models can reduce the process of coping with dialysis disease and its stressors, and lead to improved quality of life as well as the physical and mental health of these patients. These training programs are more important when facing the impact of lack of effective social support, lack of appropriate coping styles in different situations, use of emotion-focused coping styles or negative problem-focused styles, or the individual’s inability to use problem-focused and positive emotion-focused styles, resulting in debilitating illnesses associated with the stress of chronic illness and serious health threats.

## Data Availability

The datasets used and/or analyzed during the current study are in Persian and are available from the corresponding author with permission of the study group on reasonable request.

## Abbreviations

ESRD: End-Stage Renal Disease
MS: Multiple Sclerosis
CVR: Content Validity Ratio
CVI: Content Validity Index
KMO: Kaiser-Meyer-Olkin
GFI: Goodness of Fit Index
NFI: Normed Fit Index
CFI: Comparative Fit Index
RMSEA: Root Mean Square Error of Approximation
